# Purinergic Ligands as Potential Therapeutic Tools for the Treatment of Inflammation-Related Intestinal Diseases

**DOI:** 10.3389/fphar.2018.00212

**Published:** 2018-03-14

**Authors:** Diego Dal Ben, Luca Antonioli, Catia Lambertucci, Matteo Fornai, Corrado Blandizzi, Rosaria Volpini

**Affiliations:** ^1^Medicinal Chemistry Unit, School of Pharmacy, University of Camerino, Camerino, Italy; ^2^Department of Clinical and Experimental Medicine, University of Pisa, Pisa, Italy

**Keywords:** inflammation, intestinal diseases, intestinal immune system, modulators, purinergic receptors, purinergic ligands, adenosine, therapeutic tools

## Abstract

Inflammation-related intestinal diseases are a set of various conditions presenting an overactive enteric immune system. A continuous overproduction of pro-inflammatory cytokines and a decreased production of anti-inflammatory modulators are generally observed, while morpho-functional alterations of the enteric nervous system lead to intestinal secretory and motor dysfunctions. The factors at the basis of these conditions are still to be totally identified and current therapeutic strategies are aimed only at achieving and maintaining remission states, by using therapeutic tools like aminosalicylates, corticosteroids, immunomodulators, biological drugs (i.e., monoclonal antibodies), and eventually surgery. Recent reports described a key role of purinergic mediators (i.e., adenosine and its nucleotides ATP and ADP) in the regulation of the activity of immune cells and enteric nervous system, showing also that alterations of the purinergic signaling are linked to pathological conditions of the intestinal tract. These data prompted to a series of investigations to test the therapeutic potential for inflammation-related intestinal conditions of compounds able to restore or modulate an altered purinergic signaling within the gut. This review provides an overview on these investigations, describing the results of preclinical and/or clinical evaluation of compounds able to stimulate or inhibit specific P2 (i.e., P2X7) or P1 (i.e., A_2A_ or A_3_) receptor signaling and to modify the adenosine levels through the modulation of enzymes activity (i.e., Adenosine Deaminase) or nucleoside transporters. Recent developments in the field are also reported and the most promising purine-based therapeutic strategies for the treatment of inflammation-related gastrointestinal disorders are schematically summarized.

## Introduction

Inflammatory bowel diseases (IBDs) comprise Crohn’s disease and ulcerative colitis and are conditions presenting an overactive intestinal immune system. The exact etiology of these diseases is still unclear but may be related to genetic predisposition or environmental factors and is characterized by an inappropriate immune response taking to morpho-functional alterations of the host’s enteric nervous system and intestinal secretory and motor dysfunctions. A loss of balance between the production of pro-inflammatory cytokines and anti-inflammatory mediators has been observed. Current therapeutic strategies are based on anti-inflammatory agents and targeted to achieve and maintain the remission state.

It is well established that during inflammation ATP is extracellularly released, a process involving pannexins or connexins and promoted by various stimuli (Eltzschig et al., 2012; Idzko et al., 2014). Extracellular ATP (eATP) is then degraded to adenosine by the ectonucleotidases CD39 and CD73 (Allard et al., 2017). While eATP generally plays a pro-inflammatory role through the activation of P2 (P2X and P2Y) purinergic receptors, the ATP degradation to adenosine usually represents a stop-signal for the inflammation process, with adenosine playing as anti-inflammatory agent through the activation of its P1 receptor targets. Adenosine is then removed from the extracellular environment by nucleoside transporters and/or metabolic enzymes.

Over the years increasing evidences pointed out a critical involvement of the purinergic system in the pathophysiology of IBDs, thus spurring the research toward the evaluation of the potential therapeutic benefits in terms of anti-inflammatory activity, arising by pharmacological targeting of purinergic pathways (Hasko and Cronstein, 2004; Hasko and Pacher, 2008; Hasko et al., 2008; Burnstock, 2011, 2014; Burnstock et al., 2017). Furthermore, the involvement of ATP in the enteric motor dysfunctions associated with bowel inflammation is a hot topic deserving further investigations.

## P2 Purinergic Receptors

### P2X Purinergic Receptors

P2X receptors are ligand-gated ion channels activated by eATP and permeable to Na^+^, K^+^, and Ca^2+^ (North and Jarvis, 2013; North, 2016). Seven P2X subtypes are known that may assemble as homo- or heterotrimers. Upon prolonged stimulation, some subtypes like the P2X7R undergo a rearrangement with the formation of a pore permeable to large molecules.

P2XR modulators are of great interest for several potential therapeutic applications, like treatment of pain, cough, cancer, and inflammation-related diseases (Burnstock and Kennedy, 2011; Syed and Kennedy, 2012; Muller, 2015). P2XR agonists are ATP derivatives obtained by modification of the purine base (i.e., 2-meSATP), the ribose ring (i.e., BzATP), or the polyphosphate chain (like the stable analogs αβ-meATP, βγ-meATP, and ATPγS) (Coddou et al., 2011; Dal Ben et al., 2015; Lambertucci et al., 2015).

P2XR antagonists are generally negatively charged molecules like TNP-ATP (Virginio et al., 1998) and analogs (Dal Ben et al., 2017), the irreversible inhibitor oxidized ATP (o-ATP) (Murgia et al., 1993), the P2X3R antagonist A-317491, and the polyanion suramin and its derivatives. Further classes of P2XR inhibitors are uncharged molecules based on heterocyclic scaffolds and behaving as non-competitive (allosteric) antagonists (Muller, 2015). A relevant number of structural classes of compounds were developed as P2X7R inhibitors (Park and Kim, 2017) given the key role of this receptor in pain and inflammation-related conditions (Arulkumaran et al., 2011; Gulbransen et al., 2012; De Marchi et al., 2016; Burnstock and Knight, 2017; Di Virgilio et al., 2017). P2X7R-targeting compounds have been developed also as radiolabeled molecules to be used as pharmacological tools or markers (Donnelly-Roberts et al., 2009; Lord et al., 2015; Fantoni et al., 2017; Territo et al., 2017; Jin et al., 2018).

The potent, selective, and orally bioavailable P2X7R antagonist AZD9056 was studied in phase-two clinical trials for the treatment of rheumatoid arthritis (RA) and chronic obstructive pulmonary disease (COPD) showing to be well tolerated (2005-004110-32_Clinical_Trial_Results, 2005; Keystone et al., 2012). The efficacy and safety of AZD9056 was also clinically assessed in the management of patients affected by moderate/severe Crohn’s disease. Although the lack in change of inflammatory parameters, this study demonstrated that AZD9056 has the potential to improve symptoms, in particular abdominal pain, in patients with IBDs (Eser et al., 2015). Other P2X7R antagonists (CE-224,535 and GSK1482160) were studied in clinical trials for RA and inflammatory pain conditions or showed (JNJ47965567) ability to enter the CNS.

In a rat model of trinitrobenzene sulfonic acid (TNBS) colitis, the administration of the P2X7R inhibitor A740003 determined a reduction of T-cell and macrophage infiltration in the lamina propria, followed by a reduction in tissue TNF and IL-1β concentrations, with a consequent amelioration of inflammation severity (Marques et al., 2014). In parallel, Neves et al. (2014) reported that mice lacking P2X7Rs and subjected to TNBS or dextran sulfate sodium (DSS) treatment failed to develop intestinal inflammation or other symptoms associated with colitis, thus indicating a protective role resulting from P2X7R blockade. In the same study, by exploring the expression of this receptor subtype in colonic mucosa of IBD patients, the authors observed high P2X7R levels in inflamed epithelium and lamina propria, where it colocalizes more with dendritic cells and macrophages, leading to hypothesize a role of P2X7R signaling in the pathogenesis of IBDs. Furthermore, Cesaro et al. (2010) pointed out the pivotal role of P2X7R in the complex cross-talk occurring between intestinal epithelial cells and immune cells. The pharmacological stimulation of P2X7R in a human colonic epithelial cell monolayer induced caspase-1 activation and IL-1β release, pro-inflammatory mediators critically involved in the recruitment of polymorphonuclear leukocytes within the intestinal mucosa in the presence of inflammation. Subsequently, several preclinical studies performed in animal models of colitis revealed a significant role of P2X7R in the pathophysiology of intestinal inflammation (Marques et al., 2014; Neves et al., 2014; Wan et al., 2016). Increasing efforts have been made to investigate the involvement of purinergic pathways in the pathophysiology of enteric motor dysfunction typically observed in the presence of intestinal inflammation, although the available data are fragmentary (Antonioli et al., 2013). Recently, Antonioli et al. (2014b) provided evidence about a marked increase in P2X7R immunostaining, and an enhanced modulating action of these receptors on colonic neuromotility in a rat model of DNBS-induced colitis.

Beside the importance of the P2X7R in the gastrointestinal diseases described in literature, recent papers suggest that even other P2X subtypes could play a relevant role the gastrointestinal pathophysiology (Paulino et al., 2011; Weng et al., 2015; Guo et al., 2016). Antagonists of the P2X3R showed promising activity in alleviating inflammatory and neuropathic pain in preclinical studies. A-317491 reduced visceral hypersensitivity in an experimental model of colitis, suggesting P2X3R as target for the treatment of inflammation-related abdominal pain syndromes (Deiteren et al., 2015).

### P2Y Purinergic Receptors

P2Y receptors are G protein-coupled receptors of which eight subtypes (P2Y_1_, P2Y_2_, P2Y_4_, P2Y_6_, and P2Y_11-14_) are currently known. The endogenous agonists of these receptors are ATP, ADP, UTP, UDP, and UDP-glucose, with each P2Y subtype presenting peculiar pharmacological properties and preference for di- or triphosphate nucleotides (Jacobson and Muller, 2016). P2YRs are widely expressed in the body and involved in several biological functions. Beside the well-known inhibition of platelet aggregation, for which P2Y_12_R modulators like Clopidogrel, Prasugrel, Cangrelor, and Ticagrelor are approved for human use, P2YRs play important roles in neurotransmission and modulation of immune system (Le Duc et al., 2017).

P2YR ligands were developed by modification of endogenous ligands at the base (i.e., 2-thioUTP or 2-MeSATP), the sugar (i.e., MRS2365), or the phosphate chain (i.e., PSB1114) (von Kugelgen, 2006; Jacobson et al., 2012; Conroy et al., 2016; Jacobson and Muller, 2016). Dinucleoside derivatives are also ligands of the P2YRs. Diquafosol (approved in Japan for the treatment of Dry Eye disorder) and Denufosol (clinically evaluated for bronchial indication in cystic fibrosis) are representative compounds of this category and are endowed with dual P2Y_2_/P2Y_4_ receptor agonist profile. Suramin-based compounds are also P2YR modulators. Yet, several P2YR ligands belong to other structural classes not necessarily containing negatively charged functions (Conroy et al., 2016).

Considering the role of the P2Y_2_, P2Y_6_, and P2Y_12_ receptors in the inflammation-related conditions, compounds acting on these P2Y subtypes have been evaluated in such frames. P2Y_12_R antagonists present interesting potential to prevent a chronic inflammation promoted by this protein and to modulate the inflammatory pain (Thomas and Storey, 2015; Beko et al., 2017). Focusing on the bowel-related conditions, Grbic et al. (2008), investigating the role of ATP receptors in the pathogenesis of intestinal inflammation, reported that the pro-inflammatory cytokines TNF-α or IFN-γ determined an increased expression of P2Y_2_ and P2Y_6_ receptors in the colonic mucosa of mice with DSS colitis (Grbic et al., 2008; Degagne et al., 2013). Noteworthy, the pharmacological activation of P2Y_2_R via 2-thioUTP in a mouse model of DSS-induced colitis reduced the disease activity index and histological score values as well as a decrease in bacterial translocation to the spleen when compared with untreated mice, thus highlighting a protective role of P2Y_2_R in sustaining the remission phase in this experimental model of colitis (Degagne et al., 2013). By contrast, stimulation of the P2Y_6_R led to the activation of calcium-independent PKCδ upstream of ERK1/2, followed by the stimulation of c-fos phosphorylation and the recruitment of c-fos/c-jun dimers at level of the AP-1 motif located within the core promoter region of IL-8 gene, thus determining an increase of IL-8 release (Grbic et al., 2012). Despite these encouraging results, further investigations are needed to evaluate the putative beneficial effect of P2Y_2_ and P2Y_6_ receptor ligands in counteracting intestinal inflammation.

Considering the enteric motor dysfunction associated to intestinal inflammation, interesting findings were provided about the involvement of P2Y_1_R in the regulation of colonic neuromuscular activity in a model of TNBS-induced colitis in guinea-pig (Strong et al., 2010). Inflamed colonic specimens displayed a marked decrease in the fecal pellet output and a significant reduction of inhibitory junction potential (IJP). Of note, the pharmacological analysis of the determinant of IJP revealed that the purinergic component, mediated by P2Y_1_R, was impaired, despite immunohistochemical assays did not display significant alterations of nerve fiber density in circular muscle strips from animals with colitis (Strong et al., 2010).

## P1 Adenosine Receptors

Adenosine Receptors (P1 receptors or ARs) are G protein-coupled receptors known as four subtypes (A_1_AR, A_2A_AR, A_2B_AR, and A_3_AR). Like the other Purinergic Receptors, ARs are widely expressed in the body and regulate many physiological functions. The endogenous ligand adenosine has a short half-life as it is internalized by nucleoside transporters and/or quickly modified to inosine by Adenosine Deaminase (ADA) or to AMP by Adenosine Kinase (ADK).

Medicinal chemistry efforts were aimed at developing compounds presenting higher metabolic stability and improved potency and selectivity compared to the endogenous ligand (Cristalli and Volpini, 2003; Jacobson and Gao, 2006; Wilson and Mustafa, 2009; Ciruela, 2011). A key modification of adenosine is the introduction of a *N*-alkyl-carboxamido function in the 4′-position to obtain NECA (*N*-ethyl) or MECA (*N*-methyl) derivatives, with an improved potency at all the ARs. Typical A_1_AR and A_3_AR agonists contain bulky groups in the *N*^6^-position combined with modifications in the 2-position (Kim et al., 1994; Kiesman et al., 2009; Volpini et al., 2009). Reference A_3_AR agonists are IB-MECA and Cl-IB-MECA, in clinical trials for inflammation-related conditions (RA and psoriasis) (Borea et al., 2015; Jacobson et al., 2017). A_2A_AR agonists are generally NECA derivatives presenting complex chains in the 2-position (i.e., CGS21680, ATL-146e, and ATL-313 where the 4′-carboxamido group is further modified). High A_2A_AR affinity and selectivity was obtained by inserting a bulky arylalkyl function in the *N*^6^-position to obtain UK-432097 (de Lera Ruiz et al., 2014).

About the molecules able to block the AR function, AR antagonists are generally divided in non-xanthine- and xanthine-based derivatives. Non-xanthine AR antagonists are based on a large variety of scaffolds (generally heterocycles). Reference compounds for pharmacological studies at ARs may be found within this heterogeneous group, some of which were developed also as water-soluble molecules, pro-drugs, and radiolabeled compounds. Xanthine-based AR antagonists contain the A_2A_AR inhibitor Istradefylline that was approved to market in Japan as antiparkinsonian tool (Jacobson and Muller, 2016).

At present, most of available studies investigating the role of AR signaling in several experimental models of colitis showed remarkable beneficial effects upon pharmacological modulation of A_2A_AR (Odashima et al., 2005; Cavalcante et al., 2006; Naganuma et al., 2006; Rahimian et al., 2010; Antonioli et al., 2011; Pallio et al., 2016). The A_2A_AR agonists ATL-146e or ATL-313 significantly reduced mucosal inflammation of colon, with a marked decrease in pro-inflammatory cytokine levels and in leukocyte infiltration and an increase of levels of anti-inflammatory cytokines (Naganuma et al., 2006; Odashima et al., 2006). Recently Pallio et al. (2016) demonstrated the beneficial effects arising from A_2A_AR stimulation with polydeoxyribonucleotide in two experimental models of colitis. In the DSS model polydeoxyribonucleotide could counteract the hemorrhagic diarrhea, improve the weight loss, and restore the anatomic integrity of damaged epithelial and mucosal layers. In the DNBS model, polydeoxyribonucleotide markedly reduced the inflammatory response as well as the granulocytic infiltration into the mucosal and submucosal layers and, therefore, decreased the pro-inflammatory cytokines TNF and IL-1β, MPO activity and lipid peroxidation in colon samples. Noteworthy, polydeoxyribonucleotide treatment also affected Bax and Bcl-2 expression, reducing apoptotic and necrotic cells in all tissue layers. By contrast, no beneficial effects have been reported upon administration of CGS21680 in mouse model of DSS-induced colitis (Selmeczy et al., 2007). Further investigations are needed to better characterize the therapeutic potential of A_2A_AR agonists in IBDs. A number of evidences reported that aging is often associated with a chronic, low-grade systemic inflammatory condition (Laflamme et al., 2017), that could predispose to the gastrointestinal alterations typical of the elderly subject (Remond et al., 2015). Recently, it has been demonstrated that the reduction of A_2A_AR in the digestive tract of aged mice, contributes to an increased inflammation and lower ability to counteract gut infection with deleterious effects in the elderly (Rodrigues et al., 2016).

Another promising option aimed at counteracting the bowel inflammation is the pharmacological stimulation of A_3_AR (Gessi et al., 2008; Antonioli et al., 2014a). The administration of IB-MECA revealed to afford a protective role in murine models of intestinal inflammation (Mabley et al., 2003; Guzman et al., 2006). In detail, the pharmacological engagement of A_3_AR determined the inhibition of several cytokine/chemokine/inflammatory genes, thus promoting a marked down-regulation of several pro-inflammatory mediators (MIP-1α and MIP-2, IL-1, IL-6, IL-12) and the production of reactive species of oxygen, determining an improvement of the intestinal damage (Guzman et al., 2006). A recent preclinical study by Ren et al. (2015) showed that the pharmacological stimulation of A_3_AR with Cl-IB-MECA inhibited the NF-κB pathway in the colonic epithelia of DSS colitis mice. The inhibition of both NF-κB activation and IκBa phosphorylation caused a reduction of pro-inflammatory cytokines expression in colonic epithelia of inflamed animals.

Finally, the evidence supporting an over-expression of A_2B_AR in experimental colitis has sparked interest on the potential therapeutic implications of these intriguing receptor subtype (Kolachala et al., 2005). The same research group (Kolachala V. et al., 2008; Kolachala V.L. et al., 2008) demonstrated also a critical role of intestinal epithelial A_2B_AR in the pro-inflammatory activity exerted by adenosine in animals with TNBS or DSS colitis. The treatment with the A_2B_AR antagonist ATL-801 to mice with experimental colitis ameliorated several inflammatory parameters, leading to a beneficial impact on the disease progression (Kolachala V. et al., 2008). By contrast, Frick et al. (2009) reported a detrimental effect exerted by the A_2B_AR inhibitor PSB1115 in the acute phase of DSS-induced colitis, thus questioning the beneficial effect of A_2B_AR blockade in the management of IBDs.

## Regulation of Extracellular Adenosine Levels

Several authors investigated the efficacy of pharmacological treatments aimed at increasing the levels of endogenous adenosine, through the blockade of pivotal catabolic enzymes, as an alternative way to counteract intestinal inflammation. eATP is rapidly degraded to adenosine by ectonucleotidases CD73 and CD39. Genetic deletion of these enzymes prompts a higher susceptibility to inflammatory states or more severe progression of inflammation in IBD experimental models (Idzko et al., 2014; Longhi et al., 2017). Polymorphisms taking to lower ectonucleotidases expression takes to analog scenarios (Idzko et al., 2014). By contrast, blockade of nucleoside transporters leads to an increase of extracellular adenosine levels, with a consequent improvement of the inflammation course in IBD models (Ye and Rajendran, 2009). The extracellular levels of adenosine are also regulated by the activity of metabolic enzymes like ADK and ADA (Cristalli et al., 2001). The blockade of these enzymes is associated to anti-inflammatory effects and was preclinically tested as strategy to ameliorate intestinal inflammation-related conditions. Siegmund et al. (2001) reported, for the first time, an anti-inflammatory effect of the ADK inhibitor GP515 in a mouse model of colitis. Encouraging ameliorative effects were observed also following the inhibition of ADA in murine models of intestinal inflammation (Antonioli et al., 2007, 2010, 2012; La Motta et al., 2009), with reduction of the colonic inflammatory damage and decrease in tissue levels of pro-inflammatory cytokines (IL-1β, IL-6, TNF-α, IFN-γ, and chemokine C-X-C motif ligand 10), as well as a reduction of neutrophil infiltration and ROS production (Antonioli et al., 2007, 2010; Brown et al., 2008; La Motta et al., 2009). Noteworthy, it was observed that the attenuation of colonic injury, following ADA blockade, was mediated mainly by the engagement of A_2A_AR and, to a lesser extent, A_3_AR (Antonioli et al., 2010).

## Conclusion

**FIGURE 1:**
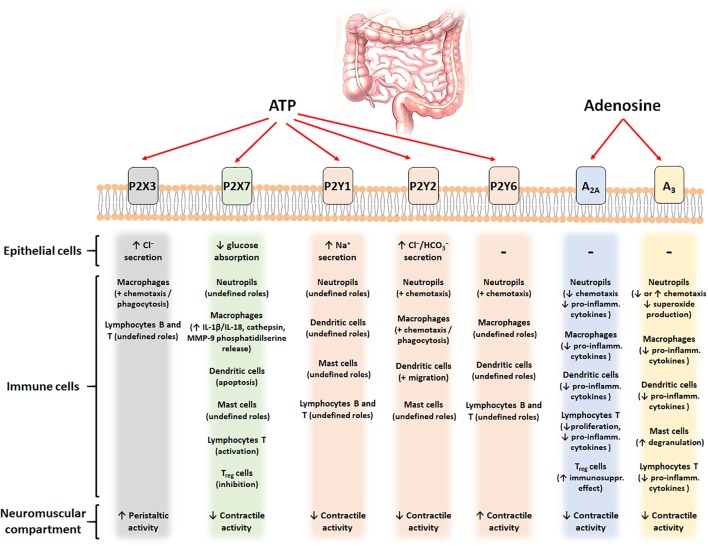
Schematic figure with the putative contribution of the main purinergic signals (ATP and adenosine) and their different receptors (P2X3, P2X7, P2Y_1,2,6_, A_2A_, and A_3_) in different cell types of the intestinal tract.

Several pre-clinical studies pointed out a key role for the purinergic system in the modulation of inflammatory and immune responses. Furthermore, clinical evaluation of purinergic ligands for the treatment of inflammation-related conditions (i.e., RA and psoriasis but also IBDs for a P2X7R inhibitor) showed a good tolerability of these molecules and suggests further investigations for this strategy. **Figure 1** and **Table 1** provide a schematic overview of the purinergic signal contributions in the intestinal tract and the most promising purine-based therapeutic strategies for the intestinal disorders.

**Table 1:** Promising pharmacological tools acting on purinergic receptors to manage intestinal disorders.

Intestinal Diseases	Pharmacological Target
Inflammatory bowel diseases (IBDs)	P2X7R antagonists
	A_2A_AR agonists
	A_2B_AR antagonists
	A_3_AR agonists
Irritable bowel syndrome (IBS)	P2X3R antagonists
	P2X7R antagonists
	A_3_AR agonists (?)
Functional motor disorders	A_1_AR antagonists (post-operative ileus)
	A_2A_AR antagonists (functional dyspepsia)
	A_2B_AR antagonists (constipation)
	A_3_AR antagonists (constipation)
Visceral pain	P2X3R antagonists
	P2X7R antagonists
	A_1_AR agonists (?)
	A_2A_AR agonists (?)
	A_3_AR agonists (?)
Diarrhea	P2X3R antagonists
	P2Y_1_R antagonists (?)
	P2Y_2_R antagonists (?)
	P2Y_6_R antagonists (?)
	A_2A_AR agonists
	A_2B_AR antagonists
	A_3_AR agonists (?)

*The (?) symbols indicate that the therapeutic potential arises from some literature contributions but is not yet clearly confirmed*.

Despite clinical studies demonstrated an encouraging profile in terms of tolerability for AZD9056, a novel P2X7R antagonist, it displayed a limited efficacy in the management of patients affected by moderate/severe Crohn’s disease. In parallel, an increasing interest has been payed toward the A_2A_AR (and the A_3_AR) agonists as viable way to manage digestive dysfunctions. However, despite promising the available evidences are limited to the pre-clinical phase and deserve further translational and clinical insights before highlighting their therapeutic potential. In this context, Michael Sitkovsky (Fredholm et al., 2007; Ohta and Sitkovsky, 2009) undoubtedly provided several of the most eminent scientific evidences about the therapeutic potential of ligands acting on this receptor subtype, paving the way toward their next clinical employment.

Apart from animal studies, the unique window of opportunity to grasp the relevance of part of the purinergic system, operated by adenosine receptors, in intestine function, is offered by the consumption of caffeine, which only known target at non-toxic conditions is the antagonism of adenosine receptors. In this regard, there are evidences, despite conflicting, about the association between caffeine consumption and the onset and development of gastrointestinal-related disorders. In particular, several authors reported a direct association between coffee consumption and some functional digestive disorders (i.e., gastro-oesophageal reflux, dyspepsia, irritable bowel syndrome) (Boekema et al., 1999; DiBaise, 2003).

These data boost the medicinal chemistry toward the synthesis of novel pharmacological entities acting selectively on specific purinergic receptors/enzymes and endowed with improved pharmacodynamic and pharmacokinetic profiles. These molecules would definitively help to clearly depict the pathophysiological role and the therapeutic potential of these proteins and would represent a key step for the development of useful tools for the management of intestinal inflammatory disorders (Burnstock, 2017a,b; Burnstock et al., 2017).

## Author Contributions

DDB and LA drafted this manuscript. DDB, LA, CL, MF, CB, and RV were responsible for idea conception, critical evaluation, and manuscript review.

## Conflict of Interest Statement

The authors declare that the research was conducted in the absence of any commercial or financial relationships that could be construed as a potential conflict of interest. The handling Editor declared a past co-authorship with one of the authors DDB.
